# Ikaros mediates the DNA methylation-independent silencing of *MCJ/DNAJC15* gene expression in macrophages

**DOI:** 10.1038/srep14692

**Published:** 2015-09-30

**Authors:** Nicolás Navasa, Itziar Martin-Ruiz, Estíbaliz Atondo, James D. Sutherland, Miguel Angel Pascual-Itoiz, Ana Carreras-González, Hooman Izadi, Julen Tomás-Cortázar, Furkan Ayaz, Natalia Martin-Martin, Iviana M Torres, Rosa Barrio, Arkaitz Carracedo, Elias R. Olivera, Mercedes Rincón, Juan Anguita

**Affiliations:** 1 Department of Veterinary and Animal Sciences. University of Massachusetts Amherst. Amherst, MA 01003; 2 CIC bioGUNE. 48160 Derio, Bizkaia, Spain; 3 Ikerbasque, Basque Foundation for Science. 48011 Bilbao, Bizkaia, Spain; 4 Biochemistry and Molecular Biology Department, University of the Basque Country (UPV/EHU), P. O. Box 644, E-48080 Bilbao, Spain; 5 Department of Molecular Biology, Veterinary School, University of León. 24071 León, Spain; 6 Department of Medicine. University of Vermont College of Medicine. Burlington, VT 05405.

## Abstract

MCJ (DNAJC15) is a mitochondrial protein that regulates the mitochondrial metabolic status of macrophages and their response to inflammatory stimuli. CpG island methylation in cancer cells constitutes the only mechanism identified for the regulation of *MCJ* gene expression. However, whether DNA methylation or transcriptional regulation mechanisms are involved in the physiological control of this gene expression in non-tumor cells remains unknown. We now demonstrate a mechanism of regulation of MCJ expression that is independent of DNA methylation. IFNγ, a protective cytokine against cardiac inflammation during Lyme borreliosis, represses MCJ transcription in macrophages. The transcriptional regulator, Ikaros, binds to the MCJ promoter in a Casein kinase II-dependent manner, and mediates the repression of *MCJ* expression. These results identify the *MCJ* gene as a transcriptional target of IFNγ and provide evidence of the dynamic adaptation of normal tissues to changes in the environment as a way to adapt metabolically to new conditions.

MCJ (Methylation-Controlled J protein), also known as DNAJC15, is a small protein (147 aa) that contains a highly conserved 70 aa J domain at the C-terminus, an unusual transmembrane domain, and an N-terminal region with no homology to any other known protein^1^,^2^,^3^. The *MCJ* gene originated as a gene-duplication from the related gene *DnaJC19*, already present in flies^2^. MCJ is located in the inner mitochondrial membrane where it interacts with Complex I of the electron transport chain (ETC), interfering with the formation of supercomplexes composed of complexes I, III and IV^4^,^5^. MCJ is the first described endogenous negative regulator of Complex I that has also been associated with the TIM23 translocase and the import of pre-proteins to the mitochondria^3^. Silencing MCJ expression does not affect cell survival or proliferation^5^. However, loss of MCJ results in augmented mitochondrial membrane potential, increased oxidative respiration and mitochondrial ATP^5^. Although MCJ deficiency has no harmful effects under physiological conditions, increased mitochondrial metabolism in the absence of MCJ *in vivo* prevents the pathological accumulation of lipids in the liver during starvation or high cholesterol diet, and the development of liver steatosis^5^. MCJ is thus a modulator of mitochondrial metabolism that acts as a break to attenuate mitochondrial metabolism during adaptation to metabolic stress conditions.

MCJ was initially identified as a gene expressed in some but not all ovarian cancer cell lines and primary ovarian cancer tumors^6^. MCJ is expressed in breast and uterine cancer cells that are sensitive to different chemotherapeutic drugs, but not in those that are multidrug resistant^2^,^6^. In normal human and murine tissues, MCJ is highly expressed in heart, liver and kidney and within the immune system, in CD8^+^ T cells and macrophages^2^,^7^. DNA methylation constitutes the only mechanism associated with the regulation of MCJ expression. In ovarian cancer cells, the presence of high levels of CpG island methylation within the first exon of the *MCJ* gene is associated with loss of expression and correlates with a diminished response to chemotherapy and poor survival^1^,^6^,^8^,^9^,^10^. However, the mechanisms that regulate MCJ expression in normal tissues and cells are not known.

We have shown that MCJ modulates macrophage responses to a variety of proinflammatory insults^7^. Short-term induction of inflammation by infection with *Staphylococcus aureus* or injection with LPS prevented TNF production *in vivo* and the development of acute fulminant hepatitis in mice in the absence of MCJ^7^. MCJ is therefore, a potential therapeutic target under conditions of persistent inflammation. Here, we report that IFNγ regulates the expression of MCJ in macrophages through a mechanism that involves the transcriptional regulator, Ikaros. These data demonstrate a novel mechanism of *MCJ* gene expression regulation that is independent of DNA methylation.

## Results and Discussion

### Loss of MCJ expression in heart-infiltrating macrophages during infection with *B. burgdorferi*

**Figure 1:**
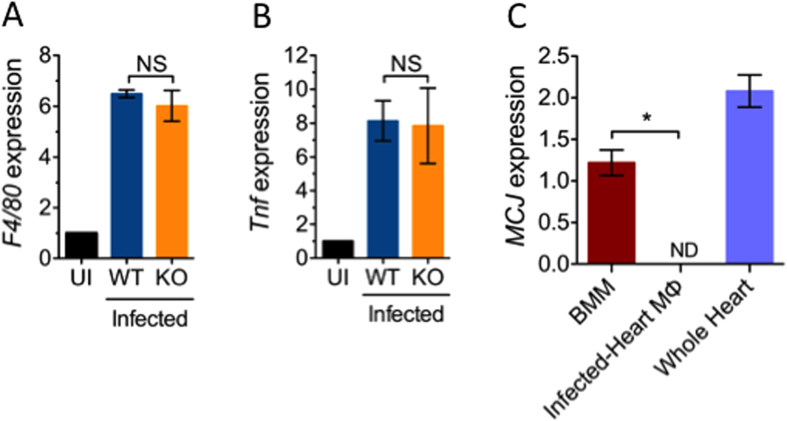
Heart-infiltrating macrophages do not express MCJ upon infection with *B. burgdorferi*. The base of the hearts of 3 week infected and uninfected (UI) mice were used to extract RNA and assess macrophage infiltration and TNF expression levels by qRT-PCR using primers specific for *F4/80* (**A**) or *TNF* (**B**). NS; Not significant. (**C**) Macrophages were purified from the hearts of 3-week infected B6 mice and used to extract RNA. qRT-PCR was then performed to detect *MCJ* mRNA levels, compared to bone marrow-derived macrophages (BMM). As a control, MCJ mRNA levels were also determined in whole heart tissue of 3-week infected mice. The data shown correspond to 5 mice per group and are presented as the mean ± SE. *; Student´s t test, p < 0.05.

During short-term *in vivo* inflammatory conditions, MCJ regulates the response of macrophages to *Staphylococcus aureus* as well as LPS treatment in mice sensitized with galactosamine 7. In order to determine the role of MCJ on the local macrophage response during an infectious process that requires a more complex and long lasting interaction between the pathogen and the host, we infected MCJ KO and WT mice with *Borrelia burgdorferi*. After 3 weeks of infection, macrophage infiltration was not significantly different in infected MCJ KO mice and WT animals (Fig. 1A). In addition, the amount of TNF expressed in the cardiac tissue upon infection was not altered in the absence or presence of MCJ (Fig. 1B). We also assessed the level of expression of MCJ in heart-infiltrating macrophages at the peak of infection with the spirochete. Surprisingly, in contrast to bone marrow-derived macrophages (BMMs), real time RT-PCR failed to detect appreciable levels of *MCJ* mRNA in macrophages infiltrating the hearts (Fig. 1C). The downregulation of MCJ expression during infection was selective of macrophages since total heart *MCJ* expression levels were readily detected in the infected mice (Fig. 1C). The histological analysis of infected joint and heart tissue showed that the degree of cardiac inflammation was not affected by the lack of the *MCJ* gene (Fig. S1A,B). Furthermore, the levels of spirochetal DNA were similar in WT and MCJ KO mice (Fig. S1C). These results suggested that upon infection with *B. burgdorferi*, *MCJ* expression is repressed specifically in macrophages infiltrating the heart.

### MCJ expression in macrophages is selectively downregulated by IFNγ

**Figure 2:**
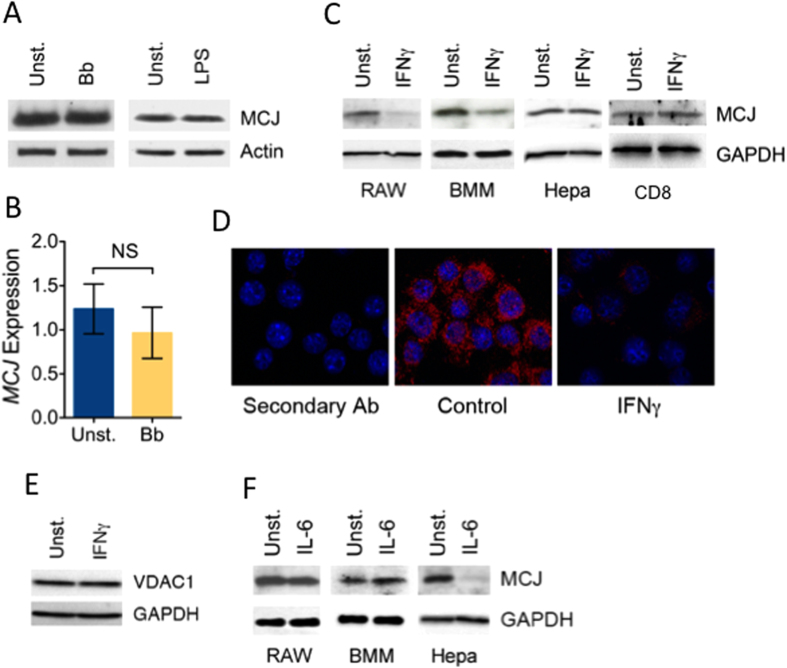
IFNγ induces the repression of MCJ in macrophages. (**A**) RAW cells were stimulated with live *B. burgdorferi* (m.o.i = 25) or 100 ng/mL of LPS for 16 h and analyzed by immunoblotting for MCJ protein levels. Actin levels were determined to ensure equal loads. (**B**) BMMs were stimulated with live *B. burgdorferi* for 16 h and analyzed for *MCJ* mRNA levels by qRT-PCR. The data shown correspond to the mean ± SE of 3 points per group. (**C**) RAW cells (RAW), BMMs, Hepa 2–7 cells (Hepa) or CD8^+^ T cells (CD8) were stimulated with 100 ng/mL of IFNγ for 24–48 h, followed by the analysis of MCJ protein levels by immunoblotting. GAPDH levels were determined to ensure equal protein loads. (**D**) RAW cells were stimulated for 72 h with 100 ng/mL of IFNγ in 8-well chamber slides, washed and stained for intracellular MCJ. The slides were analyzed by ApoTome fluorescence microscopy. (**E**) RAW cells stimulated with IFNγ were analyzed for the levels of the mitochondrial protein, VDAC1, by immunoblotting. (**F**) RAW, BMMs and Hepa cells were stimulated with 100 ng/mL of IL-6 for 24 h, followed by their analysis for MCJ protein content by immunoblotting.

In order to determine whether the interaction of macrophages with bacterial products results in reduced levels of MCJ, we stimulated RAW cells and BMMs with live *B. burgdorferi* and assessed the levels of MCJ. Stimulation with the spirochete did not affect MCJ protein (Fig. 2A) or mRNA (Fig. 2B) levels. LPS stimulation also failed to alter the levels of MCJ in macrophages (Fig. 2A). These data indicate that the regulation of the expression of MCJ occurs independently of pattern-recognition receptor (PRR) stimulation, including TLR4, TLR1/2 and other PRRs stimulated by the interaction of live *B. burgdorferi* with macrophages^11^,^12^,^13^,^14^,^15^. Since IFNγ is a major contributor to macrophage function during cardiac infection with *B. burgdorferi*^16^,^17^, we stimulated macrophages with IFNγ. Treatment with IFNγ resulted in lower levels of MCJ protein in both RAW cells and BMMs (Fig. 2C,D). Because MCJ is localized in mitochondria, we examined the effect of IFNγ on mitochondrial mass; however, no difference was observed as determined by levels of the mitochondrial protein, VDAC1 (Fig. 2E). The effect of IFNγ was selective of macrophages, because it did not affect MCJ levels in the murine tumor cell line, Hepa 1–6 or primary CD8^+^ T cells (Fig. 2C). IL-6 has been shown to downregulate MCJ levels in breast cancer cell lines^2^. Similarly, we found that IL-6 induced the downregulation of MCJ in Hepa liver cancer cells (Fig. 2F). However, IL-6 failed to downregulate MCJ expression in RAW cells or BMMs (Fig. 2F). These results show that *MCJ* expression in macrophages is selectively silenced by IFNγ.

### IFNγ inhibits *MCJ* gene transcription independently of DNA methylation

**Figure 3:**
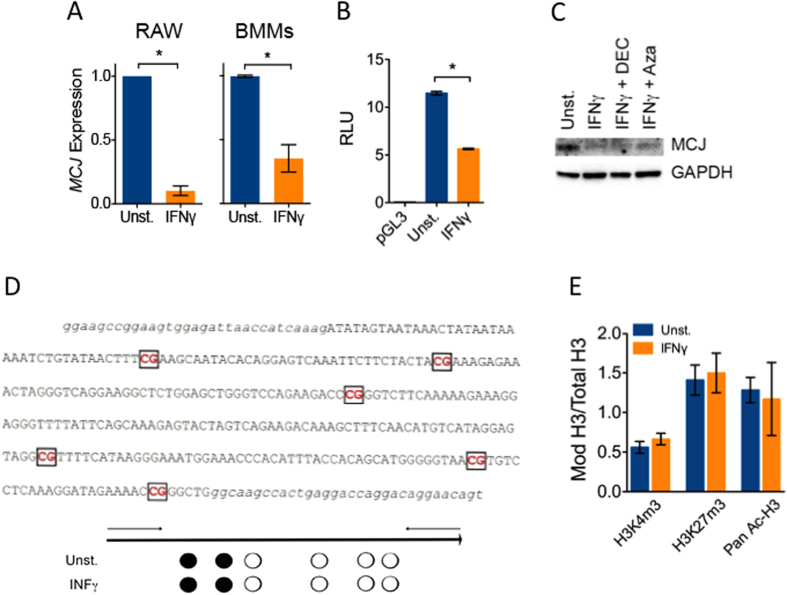
IFNγ represses *MCJ* gene expression independently of DNA methylation. (**A**) RAW cells and BMMs were stimulated for 20 h with IFNγ and analyzed by qRT-PCR for *MCJ* mRNA levels. The results correspond to the average of 3 independent experiments. *; Student´s t test, p < 0.05. (**B**) RAW cells were co-transfected with plasmids containing the luciferase gene under the influence of the 1 kb proximal promoter region of the *MCJ* gene or the Renilla luciferase gene under the influence of the SV40 promoter. After 4 h, the cells were stimulated with 100 ng/mL of IFNγ or left unstimulated. Dual luciferase activity was assessed after 16 h of incubation. The promoterless vector, pGL3 was used as a control. *; Student´s t test, p < 0.05. (**C**) BMMs were left unstimulated or stimulated with 100 ng/mL of IFNγ in the absence or presence of 1 μM of decitabine (DEC) or Azacitidine (Aza). After 48 h, the cells were tested by Western blotting for the presence of MCJ. GAPDH levels were determined to ensure equal loading. (**D**) CpG-rich region in the MCJ gene analyzed by bisulfite sequencing. The primers used for amplification are noted in lower case. The percentage of methylated CpG residues in BMMs stimulated with 100 ng/mL of IFNγ or left untreated is marked in each of the 6 CpG residues. Black circles indicate 100% of the samples contained these residues methylated, while white circles represent 0%. The analysis corresponds to BMMs isolated from 6 mice. (**E**) CHIP analysis of BMM DNA immunoprecipitated with antibodies against the H3 marks corresponding to trimethylation of Lys 4 (H3K4m3) and 27 (H3K27m3) or H3 pan-acetylation (Pan Ac-H3). The binding leves are relative to total H3. The results correspond to the average ± SE of 3 independent experiments.

To determine if the downregulation of MCJ protein levels by IFNγ in macrophages was due to an effect on MCJ gene expression, we assessed *MCJ* mRNA levels in macrophages stimulated with IFNγ. The treatment with IFNγ resulted in a significant decrease in *MCJ* mRNA levels in RAW cells and BMMs (Fig. 3A). No previous studies have characterized the human or mouse *MCJ* gene promoter region and addressed transcriptional regulation. We identified a 1 kb region upstream of the start initiation site of the murine *MCJ* gene (Fig. S2A), that was capable to mediate high levels of transcription in RAW cells in luciferase reporter assays (Fig. 3B). Treatment with IFNγ caused a pronounced decrease in the transcriptional activity of this region of the *MCJ* promoter (Fig. 3B).

The only described mechanism of regulation of *MCJ* involves the methylation of CpG rich regions of the gene^9^. Thus, we addressed whether IFNγ could silence *MCJ* expression through DNA methylation. BMMs were treated with IFNγ in the presence of the methylation inhibitors, decitabine (DEC) and 5-azacitidine (Aza). Both DEC and Aza failed to prevent the downregulation of *MCJ* expression by IFNγ (Fig. 3C). We also analyzed by bisulfite sequencing the methylation status of CpG residues present in the gene region identified as distinctively methylated between CD8^+^ T and B cells^18^ and that correlates with the level of expression of MCJ in these cells^5^,^7^. Six CpG residues were identified in this region (Fig. 3D). Of these, the first two were methylated in 100% of the BMMs samples analyzed (Fig. 3D). Importantly, the stimulation of BMMs with IFNγ did not affect the methylation of these CpG residues (Fig. 3D), indicating that IFNγ effect is independent of DNA methylation mechanisms. We further analyzed the effect of IFNγ treatment on histone H3 marks associated with the activation and repression of gene expression (ref. 31). The treatment of BMMs with IFNγ did not affect the binding of thrimethylated H3 at Lys 4 and Lys 27 or acetylated H3 to the *MCJ* promoter (Fig. 3E). These data revealed that transcriptional regulation is an alternative mechanism of *MCJ* expression modulation that is independent of DNA methylation or alteration on histone marks.

### Ikaros is an inducible repressor of MCJ gene transcription

**Figure 4:**
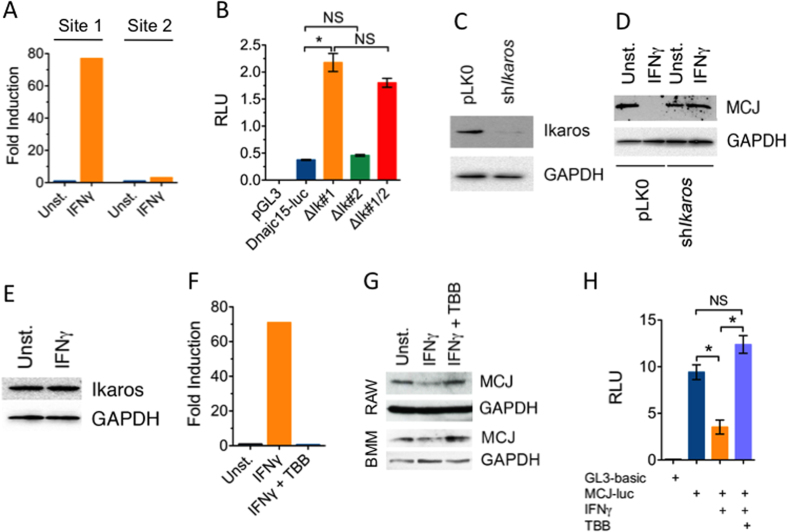
IFNγ induces the binding of Ikaros to the *MCJ* promoter through the activation of CK2. (**A**) Chromatin immunoprecipitation using an antibody specific for Ikaros. BMMs were stimulated with 100 ng/mL of IFNγ for 16 h or left unstimulated. Chromatin was processed and immunoprecipitated as described in Experimental Procedures. The values presented correspond to one experiment of 2 with similar results. (**B**) Luciferase activity driven by the 1 kb MCJ proximal promoter (MCJ-luc) and deletion mutants corresponding to Site 1 (ΔIk#1), Site 2 (ΔIk#2) or double mutants (ΔIK#1/2). The values correspond to the mean ± SE of triplicates and represent at least 3 independent experiments. *; Student´s T test, p < 0.05; NS; not significant. (**C**) Western blot showing silencing of Ikaros in RAW cells stably transduced with lentiviral particles containing a short hairpin (sh) specific for the Ikaros gene (sh*Ikzf1*). GAPDH levels were determined to ensure equal loads. (**D**) sh*Ikaros* cells and pLK0-transduced control cells were stimulated with 100 ng/mL of IFNγ or left stimulated for 24 h. The levels of MCJ were then determined by immunoblotting. (**E**) Levels of Ikaros in BMMs unstimulated or stimulated with 100 ng/mL of IFNγ for 24 h. The cells were l tested by immunoblotting using specific Ikaros antibodies. GAPDH levels were determined to ensure equal loads. (**F**) BMMs were stimulated with IFNγ in the presence or absence of TBB as before, and Ikaros binding was determined by chromatin immunoprecipitation. The values correspond to 1 of 2 experiments performed with similar results. (**G**) RAW cells and BMMs were stimulated with 100 ng/mL of IFNγ in the presence or absence of 50 μM of the CK2 inhibitor, 4,5,6,7-Tetrabromobenzotriazole (TBB), for 24 h, followed by MCJ protein level determination by immunoblotting. (**G**) RAW cells were co-transfected with the plasmids pGL3-MCJ-Luc plus pSV40-RenillaLuc. Four h later, the cells were stimulated with 100 ng/mL of IFNγ in the presence or absence of 50 μM TBB and 16 h later, assessed for luciferase levels. The values correspond to luciferase activity relative to Renilla luciferase in triplicate (mean ± SE) and represent one of at least 4 experiments performed. *; Student´s T test, p < 0.05; NS; not significant.

To identify the specific mechanism by which IFNγ represses *MCJ* gene transcription, we performed a search for potential transcription factor binding sites within the 1kb region of the mouse *MCJ* gene promoter using the tool TFSearch^19^. Two consensus binding sites for Ikaros (−350 to −361 and −706 to −717) were identified (Fig. S2A). Ikaros is known to act primarily as a repressor of gene expression^20^. To demonstrate whether Ikaros binds to these putative binding sites in the *MCJ* promoter and address whether binding was regulated by IFNγ, we performed chromatin immunoprecipitation (ChIP) assays in BMMs. Binding of Ikaros to both sites was almost undetectable in untreated BMMs (Fig. 4A). However, Ikaros binding to Site 1 (the most proximal to the transcription start site; Fig. S2A) was highly induced in cells treated with IFNγ (Fig. 4A). Ikaros binding to Site 2 (Fig. S2A), however, was not induced by IFNγ (Fig. 4A).

To further analyze the contribution of both putative Ikaros binding sites to the regulation of MCJ gene expression, we generated deletion mutants of both binding sites, as well as a double deletion mutant lacking both binding sites. The deletion of Site 1 resulted in significantly increased transcriptional activation in reporter assays (Fig. 4B) suggesting that this site is bound under basal conditions to a negative gene expression regulator. However, the deletion of Site 2 did not affect the expression activity of the promoter (Fig. 4B). Furthermore, the deletion of both sites resulted in transcriptional activity that was equivalent to that observed with the deletion of Site 1 (Fig. 4B). Overall, these data suggest that Ikaros binds to Site 1 upon induction with IFNγ and displaces a weaker negative regulator of gene expression.

To demonstrate the role of Ikaros in the IFNγ-dependent repression of *MCJ* gene expression, we generated stable lentiviral transductants containing a short hairpin sequence (shRNA) specific for the *IKFZ1* gene encoding Ikaros. Transduction with sh*IKFZ1* in RAW cells caused a prominent reduction of Ikaros levels (Fig. 4C). Importantly, while IFNγ downregulated MCJ levels in control cells, it did not affect MCJ levels in sh*IKFZ1-*transduced cells (Fig. 4D). These results demonstrate that silencing of MCJ expression by IFNγ is mediated by Ikaros and reveal this repressor as a key factor in the alternative mechanism regulating MCJ expression.

We then assessed whether IFNγ upregulates Ikaros expression. The stimulation with IFNγ did not affect Ikaros levels in macrophages (Fig. 4E), suggesting that the increased binding to the *MCJ* promoter region was due to post-translational modifications induced by IFNγ. Ikaros activity is regulated by phosphorylation mediated by Casein Kinase 2 (CK2)^21^. It has also been reported that IFNγ regulates the expression of a subset of genes through the activation of CK2^21^,^22^. To investigate whether IFNγ promotes Ikaros binding through CK2, cells were treated with IFNγ in the presence of a CK2 specific inhibitor, 4,5,6,7-tetrabromobenzotriazole (TBB)^23^. CHIP analysis demonstrated that the pretreatment with TBB abrogated IFNγ-induced binding of Ikaros to the *MCJ* promoter (Fig. 4F).

We then investigated whether silencing of MCJ expression by IFNγ was mediated by CK2. Pretreatment of macrophages with the CK2 inhibitor prevented downregulation of MCJ expression by IFNγ (Fig. 4G). In addition, inhibition of CK2 also prevented IFNγ from suppressing *MCJ* promoter transcriptional activity (Fig. 4H). Together, these results show that IFNγ represses *MCJ* gene transcription in macrophages by promoting CK2-dependent DNA binding of Ikaros to the proximal region of the MCJ promoter.

Our studies identify a novel mechanism of regulation of *MCJ* gene expression that is independent of the well-established DNA methylation pathway described in several tumors. MCJ/DnaJC15 is emerging as an important regulator of mitochondrial activity and cellular function *in vitro* and *in vivo*^5^,^7^. Therefore, the control of MCJ transcription constitutes a mechanism to regulate cellular responses to environmental changes. As opposed to DNA methylation, which is considered a long-term mechanism to silence gene expression^24^, the transcriptional control of *MCJ* gene expression by Ikaros may allow normal tissues to adapt dynamically to a changing environment. Here, we demonstrate that Ikaros represses *MCJ* expression in response to IFNγ in macrophages. Similar mechanisms could be used to alter MCJ levels in other cells or tissues in response to changes in the environment as a way to adapt metabolically to new conditions.

Our results identify *MCJ* gene expression as a transcriptional target of the cytokine IFNγ, contributing to the regulation of their inflammatory output^7^. Our data also reinforces the role of IFNγ as a cytokine that exerts a protective effect during infection with *B. burgdorferi*^16^,^17^,^25^. Overall, we hypothesize that the combined effect of IFNγ, including the regulation of MCJ expression, results in a more efficient elimination of the bacteria from the infected tissue without a concomitant increase in the inflammatory damage.

## Methods

### Mice

MCJ-deficient mice in a C57Bl/6 (B6) background^5^ and wild type B6 mice were bred at UMass Amherst and CIC bioGUNE. The Institutional Animal Care and Use Committees at UMass Amherst and CIC bioGUNE approved all procedures involving animals.

### Infections

Groups of WT and KO mice were infected by subcutaneous injection with 10^5^ *Borrelia burgdorferi* 297 in the midline of the back. The mice were sacrificed after 3 weeks of infection and analyzed for inflammatory symptoms in joints and hearts stained with hematoxilin and eosin. Signs of arthritis and carditis were determined blindly as described^26^. The number of spirochetes in heart tissue was determined by real-time PCR, using primers specific for the *recA* gene (Table S1) standardized to μg of total DNA with primers corresponding to Glyceraldehyde 3-Phosphate Dehydrogenase, *GAPDH*, (Table S1)^17^.

### Cells

Infiltrating cardiac macrophages were isolated from 3-week infected B6 mice. Hearts were perfused with cold Hank´s balanced salt solution (HBSS, Lonza, Anaheim, CA) and cut into small pieces, followed by digestion with 1 mg/mL of collagenase/dispase (Roche) and homogenization in a Dounce homogenizer. The digest was passaged through a 16” gauge syringe to obtain single cell suspensions. The cellular suspension was layered on top of a 3 mL layer of Ficoll (GE Healthcare, Piscataway, NJ) and centrifuged at 400 × g for 40 min without brakes. Monocytes were then purified from the interphasic cellular fraction using a one-step discontinuous Percoll gradient (46%) under isosmotic conditions^27^. Monocytes were used for RNA extraction.

Bone marrow-derived macrophages were generated as described^17^ using 30 ng/mL of M-CSF (Miltenyi Biotec, Bergisch Gladbach, GE). Macrophages were allowed to differentiate in 100 mm × 15 mm petri dishes (Fisher Scientific, Pittsburgh, PA) for 8 days. Non-adherent cells were then eliminated and adherent macrophages were scraped, counted and resuspended in serum-free RPMI medium 2 h prior to use.

CD8^+^ T cells were purified by positive selection from the spleens of B6 mice using biotinylated anti-CD8 (BD Biosciences, San Diego, CA), anti-biotin microbeads and the MACS system (Miltenyi Biotec, Auburn, CA).

Lentiviral particles containing shRNA targeting Ikaros (*Ikzf1* gene, Sigma Chemical Co, St. Louis, MO) were produced as described^28^. Supernatants containing the virus were used to infect RAW 264.7 cells, followed by incubation with puromycin at 2 μg/mL to generate stable lines. Cells containing the empty vector, pLK0.1, were used as a control.

### *In vitro* stimulation

Cells were incubated with 100 ng/mL of murine IFNγ or human IL-6 for the indicated time periods. In some instances, the following inhibitors were used 1 h prior to stimulation: decitabine (DEC, 1mM), 4,5,6,7-tetrabromobenzotriazole (TBB, 1 μM; Tocris Bioscience, Bristol, UK), 5-Aza-2-Deoxycytidine (Aza, 1 μM; Sigma Chemical Co.). Stimulations with *B. burgdorferi* (m.o.i. = 25) or LPS (100 ng/mL) were performed for 4–6 h.

### Real-time RT-PCR

RNA from isolated cells or cardiac tissue was extracted by the thioisocyanate method (Amresco, Solon, OH), treated with DNase I (Qiagen), and reverse transcribed using the SuperScript VILO cDNA synthesis kit (Life Technologies). Real-time PCR was then performed using SYBR Green PCR Master Mix (Life Technologies) on a BioRad CFX96 Real-Time System (Bio-Rad, Hercules, CA). Fold induction of the genes was calculated relative to actin, using the 2^−ΔΔCt^ method. The primers used are listed in Table S1.

### Western blot

Five to 20 μg of protein were run on SDS-PAGE, transferred to nitrocellulose membranes and tested with antibodies specific for MCJ^5^, VDAC1 (D-16) and Ikaros (M-20, Santa Cruz Biotechnology, Dallas, TX). Equal loading was determined using antibodies against GAPDH (6C5) or actin (I-19) from Santa Cruz Biotechnology.

### Epifluorescence (Apotome) microscopy

Cells were grown in 8-well chamber slides (Nunc Thermo Scientific, Waltham, MA). Upon incubation with 100 ng/mL of IFNγ (eBioScience, San Diego, CA) for 3 days, the cells were processed as described^29^ using anti-MCJ Abs, followed by an anti-rabbit IgG conjugated to Alexa Fluor 594.

### Cloning of the proximal 1 kb MCJ promoter and luciferase assays

The proximal 1 kb promoter of the murine *MCJ* gene was cloned into pGL3 using the primers in Table S1. Deletion mutants corresponding to the putative Ikaros binding sites of the *MCJ* promoter (Fig. S2) were generated using the QuickChange Site-Directed Mutagenesis kit (Stratagene, La Jolla, CA) and the primers listed in Table S1. 1.9 μg of these constructs plus 0.1 μg of pSVL40 plasmid were cotransfected into RAW cells using the X-TremeGene HP DNA tranfection reagent (Roche). After 6h, the cells were treated with IFNγ in the presence or absence of the specific inhibitor, TBB. After 20 hr incubation, the cells were lysed in lysis buffer (Promega, Madison, WI) and Firefly and Renilla luciferase activities were determined by the Dual Luciferase reporter system (Promega).

### Bisulfite sequencing

DNA was extracted from BMMs treated with IFNγ and controls, denatured and subjected to bisulphite conversion as described by Clark and colleagues^30^. The resultant product was PCR amplified using the primers in Table S1, corresponding to the region in the *MCJ* gene described by Meissner and colleagues^18^.

### Chromatin immunoprecipitation

Fifteen million BMMs were stimulated with 100 ng/mL of IFNγ in the presence or absence of TBB for 16h. CHIP assays were performed using the SimpleChip Enzymatic Chromatin IP kit-Magnetic beads (Cell Signaling, Beverly, MA) following the manufacturer´s instructions using anti-Ikaros, anti-H3K4m3, anti-H3K27m3, anti-pan acetylated H3 antibodies and anti-H3 (Cell Signaling) or normal rabbit IgG as negative control. The immunoprecipitated DNA was subjected to q-PCR using primers encompassing the two putative Ikaros binding sites (Table S1). The results are presented as fold induction over rabbit IgG immunoprecipitates or total H3 relative to input (percent input method), following the formula: 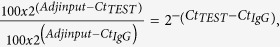 where AdjInput = Adjusted input to 100%; Ct_TEST_ = Ct of test samples; Ct_IgG_ = Ct of samples control.

### Statistical Analysis

Results are presented as means ± SE. Significant differences between means were calculated with the Student’s t test. P values of 0.05 or less were considered statistically significant.

## Additional Information

**How to cite this article**: Navasa, N. *et al.* Ikaros mediates the DNA methylation-independent silencing of MCJ/DNAJC15 gene expression in macrophages. *Sci. Rep.* **5**, 14692; doi: 10.1038/srep14692 (2015).

## Supplementary Material

Supplementary Information
