# Supplementary material for: Ikaros mediates the DNA methylation-independent silencing of MCJ/DNAJC15 gene expression in macrophages
Source: Sci Rep. 2015 Sep 30;5:14692. doi: 10.1038/srep14692 (PMC4588509; doi:10.1038/srep14692)
Supplement: Supplementary Information [file srep14692-s1.pdf]

Ikaros mediates the DNA methylation-independent silencing of *MCJ/DNAJC15* gene expression in macrophages

Nicolás Navasa [1,2], Itziar Martin-Ruiz [2], Estíbaliz Atondo [2], James D. Sutherland [2], Miguel Angel Pascual-Itoiz [2], Ana Carreras-González [2], Hooman Izadi [1], Julen Tomás-Cortázar [2], Furkan Ayaz [1], Natalia Martin-Martin [2], Iviana M Torres [1], Rosa Barrio [2], Arkaitz Carracedo [2,3,4], Elias R. Olivera [5], Mercedes Rincón [6] and Juan Anguita [1,2,3,*]

[1] Department of Veterinary and Animal Sciences. University of Massachusetts Amherst. Amherst, MA 01003.

[2] CIC bioGUNE. 48160 Derio, Bizkaia, Spain.

[3] Ikerbasque, Basque Foundation for Science. 48011 Bilbao, Bizkaia, Spain.

[4] Biochemistry and Molecular Biology Department, University of the Basque Country (UPV/EHU), P. O. Box 644, E-48080 Bilbao, Spain.

[5] Department of Molecular Biology, Veterinary School, University of León. 24071 León, Spain.

[6] Department of Medicine. University of Vermont College of Medicine. Burlington, VT 05405.

Supplementary Information

A

	Arthritis (Average ± SE)	Carditis (Average ± SE)
WT	0.6 ± 0.4	0.9 ± 0.2
MCJ KO	0.8 ± 0.2	0.8 ± 0.1

B

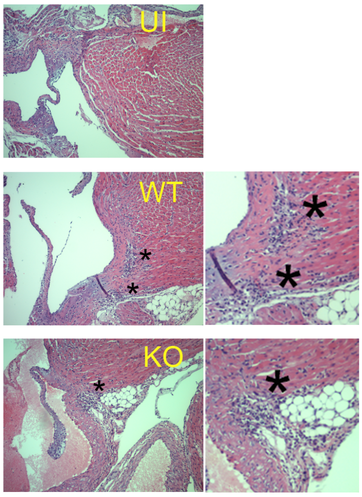

C

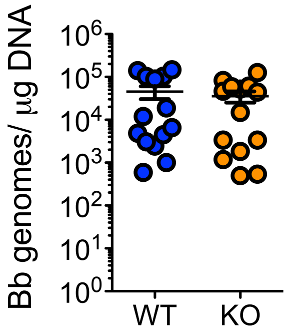

Figure S1. MCJ KO mice develop similar levels of inflammation than WT controls upon infection with *B. burgdorferi*. (A) Levels of arthritis and carditis ± SE in 3 week infected MCJ KO and WT control mice. The mice, in groups of 5, were infected with 10^5 *B. burgdorferi* subcutaneously. Arthritis and carditis were determined blindly in tissue sections processed as described in Methods. (B) Photomicrographs showing representative cardiac sections of uninfected (UI) and infected wild type (WT) or MCJ KO mice. Foci of inflammation are marked by asterisks. (C) Total DNA was extracted from the base of the heart of uninfected or WT and MCJ KO infected mice and spirochetal burdens were determined by real-time PCR as described in Methods. The results represent the mean of 5-15 mice in 3 different experiments.

*(-1000)

AATCTAGCAT GCTTGCTCGT GCTTTTAGTA TATGGTACTA GCATGGTGTT TAATGTAATC
TTTCCTTCCT GATTCTAAAT TCCTTCAGGC CCTAAGCATG AGAATAATAA TATATGGATA
AATTAATAGG TTCTTACAAC TGGTTTTTTG TTTGTTTGTT TGTATGGTTT GTTTTGTTTT
GTTTTTCAAG ATAGGGTTT**C TCTGTATAGC TCTGGCTGTC** CTGGAACTCA CTTTGTAGAC
CAGGCTGGCC TCGAACTCAG AAATCTGCCT GCCTCTGCCT CCTGGGTGCT GGGATTAAAG Ikaros #2
GCGTGCGCCA TCACGCCCAG CTAGGTTCTT ACAACTTTTA ATGTAAAAAA ACAAAACAAA
ACAAAAAAAC CAAACCCCAA AACAAAAAAA AACCTT**CTTG TAGGCTGGAT TGCAAAGTG**T
ATAAAGGGTA AAACAGCAGC CAACTTGGTA CAGGGGTTAG AAAGCGTATT AGCTTCAGTT
ATTGCCTAAT ATCTGTTCGA GAAAGCTTGA CCTAAACATT AATAATGAAG TTAAGTCTTT
ATGTATTCAA ATTAGTCAAA A**TCATTTGCT GTGAGCGCAA G**TTAGTGGAT CATCAGTATC
TTCCCAGTGA TTTCACTAAC ATGTGAAATC ACATTCATTT CTAGTTGGGA AGGTCAAGCA Ikaros #1
AGGAAGCCCT CCCTGGCCTT GTTTCTATTC CCAGGCACCA TCGCGGTACT TTGGAATCTC
TCCCTGCCTG GTTTTAGAGG AAAC**TCAAGG TAGACCTAAG AAGGC**CCCGC CTCTTCAGGC
TTGGTTGTTA TAGTGGAGGG TGGCACAAAC AAACAAACAA GGCTCTGTAA GGGTCTGACC
CCGGGCTTTG ACAGGGTCAG TTTATCGGAG ATGGAATGAG AACCAGATTC TCTGCAGCCA
GGTCTCCACT AATAAAACTG AACACCTACT CAGAGCGCGA ACTTAACCGG TCCGCACTAA
TTTGTCCCCG CGGACGCCCC **GTAGACAACT CAGTGCGTCC TCG**

+1

Figure S2. Identification of putative Ikaros binding sites on the MCJ promoter. Sequence of the 1kb proximal MCJ promoter identifying 2 Ikaros binding sites (in green). The primers used to clone the promoter are shown in red. The primers utilized for the amplification of immunoprecipitated chromating are marked in bold at both sides of each binding site (Table S1).

Table S1. Primers used and their purpose.

NAME	SEQUENCE	PURPOSE (E = efficiency)
MCJ_PROM_F (*Kpn*I)	5’-TTA A*GG TAC C*AA TCT AGC ATG CTT GCT CGT-3’	*MCJ* promoter cloning
MCJ_PROM_R (*Xho*I)	5’-AAT T*CT CGA G*CG AGG ACG CAC TGA GTT GTC TAC-3’	
CHIP_IK#1_F	5’-TCA TTT GCT GTG AGC GCA AG-3’	q-PCR for Ikaros site #1 E = 95%
CHIP_IK#1_R	5’-GCC TTC TTA GGT CTA CCT TGA-3’	
CHIP_IK#2_F	5’-CTC TGT ATA GCT CTG GCT GTC-3’	q-PCR for Ikaros site #2 E = 96%
CHIP_IK#2_R	5’-CAC TTT GCA ATC CAG CCT ACA AG-3’	
Site #1 deletion_F	5'-GTG AAA TCA CAT TCA TTT CTC AAG CAA GGA AGC CCT CCC-3'	Deletion of Ikaros Site 1 on *MCJ* promoter
Site #1 deletion_R	5'-GGG AGG GCT TCC TTG CTT GAG AAA TGA ATG TGA TTT CAC-3'	
Site #2 deletion_F	5'-GCC TGC CTC TGC CTC CTG GGA GGC GTG CGC CAT CAC GCC C-3'	Deletion of Ikaros Site 2 on *MCJ* promoter
Site #2 deletion_R	5'-GGG CGT GAT GGC GCA CGC CTC CCA GGA GGC AGA GGC AGG C-3'	
MCJ_F	5´-ACG CCG ACA TCG ACC ACA CAG-3´	qRT-PCR E = 105%
MCJ_R	5´-AAT CTT CCT TGC TGT TGC CGT G-3´	
ACTIN_F	5´-GAC GAT GCT CCC CGG GCT-3´	qRT-PCR E = 98%
ACTIN_R	5´-TCT CTT GCT CTG GGC CTC-3´	
TNF_F	5´-AGC CCA CGT CGT AGC AAA CCA C-3´	qRT-PCR E = 101%
TNF_R	5´-ATC GGC TGG CAC CAC TAG TTG GT-3´	
F4/80_F	5´-CTG CGC AGA TGT TGA TGA GTG TC-3´	qRT-PCR E = 98%
F4/80_R	5´-GGA GCC ATT CAA GAC AAA GCC TG-3´	
RecA_F	5’-GTG GAT CTA TTG TAT TAG ATG AGG CTC TCG-3’	*B. burgdorferi* quantification
RecA_R	5’-GCC AAA GTT CTG CAA CAT TAA CAC CTA AAG-3’	
GAPDH_F	5’-CCA TCA CCA TCT TCC AGG AGC GAG-3’	
GAPDH_R	5’-CAC AGT CTT CTG GGT GGC AGT GAT-3’	
